# OLDER WOMEN WITHOUT AND WITH DIABETES MELLITUS: RISK OF FALLS

**DOI:** 10.1093/geroni/igad104.2279

**Published:** 2023-12-21

**Authors:** Alessandro Oliveira de Silva, Silvana Funghetto, Luciano Ramos de Lima, Marina Morato Stival

**Affiliations:** Center University of Brasilia, Brasília, Distrito Federal, Brazil; University of Brasilia- Campus Ceilândia- UNB-Brasil, Brasília, Distrito Federal, Brazil; University of Brasilia, Brasilia, Distrito Federal, Brazil; University of Brasilia- Campus Ceilândia- UNB-Brasil,, Brasília, Distrito Federal, Brazil

## Abstract

Evidence points to an association between hyperglycemic state and worsening mobility, with increased risk of falls. The aim of this study was to compare the risk of falls in the women older with and without diabetes mellitus in a sample of women older 60 to 65 years of age (N = 205) care in primary health care in the Federal District - Brazil. The results of the statistical model showed that the highest prevalence of falls was in the group of older women with type 2 diabetes mellitus (52.8%). It was possible to verify in the Time Up and Go performance test that most older women with type 2 diabetes mellitus were at risk of falling (79.2%) (p=0.003). Furthermore, women with type 2 diabetes mellitus showed a higher risk of falls in the Berg Balance test (51.76±5.87). The results suggest that type 2 diabetes mellitus significantly increased the risk of falls in this population. The potential benefits of this approach contribute to the planning of strategies and interventions to prevent falls in this population of older women.

